# How Segregation Makes Us Fat: Food Behaviors and Food Environment as Mediators of the Relationship Between Residential Segregation and Individual Body Mass Index

**DOI:** 10.3389/fpubh.2018.00092

**Published:** 2018-03-29

**Authors:** Melody Goodman, Sarah Lyons, Lorraine T. Dean, Cassandra Arroyo, James Aaron Hipp

**Affiliations:** ^1^Department of Biostatistics, College of Global Public Health, New York University, New York, NY, United States; ^2^Division of Public Health Sciences, Department of Surgery, Washington University in St. Louis, St. Louis, MO, United States; ^3^Department of Epidemiology, Bloomberg School of Public Health, Johns Hopkins, Baltimore, MD, United States; ^4^Department of Research Patient Care Services, Barnes-Jewish Hospital, St Louis, MO, United States; ^5^North Carolina State University, Raleigh, NC, United States

**Keywords:** Residential segregation, body mass index, food envrionment, Health Behavior, Mediation

## Abstract

**Objectives:**

Racial residential segregation affects food landscapes that dictate residents’ food environments and is associated with obesity risk factors, including individual dietary patterns and behaviors. We examine if food behaviors and environments mediate the association between segregation and body mass index (BMI).

**Methods:**

Non-Hispanic Whites and Blacks living in the St. Louis and Kansas City metro regions from 2012 to 2013 were surveyed on dietary behaviors, food environment, and BMI (*n* = 1,412). These data were combined with the CDC’s modified retail food environment index and 2012 American Community Survey data to calculate racial segregation using various evenness and exposure indices. Multi-level mediation analyses were conducted to determine if dietary behavior and food environment mediate the association between racial residential segregation and individual BMI.

**Results:**

The positive association between racial segregation and individual BMI is partially mediated by dietary behaviors and fully mediated by food environments.

**Conclusion:**

Racial segregation (evenness and exposure) is associated with BMI, mediated by dietary behaviors and food environment. Elements of the food environment, which form the context for dietary behaviors, are potential targets for interventions to reduce obesity in residentially segregated areas.

## Introduction

The prevalence of obesity in the United States has risen steadily across all demographic groups but is disproportionately prevalent among Black Americans. Estimates suggest that Non-Hispanic Blacks have the highest age-adjusted rates of obesity (48%) compared to other racial/ethnic groups (1, 2). The prevalence of obesity is higher in communities with higher proportions of Black residents when compared to neighborhoods in which the majority of residents are White (3). Regardless of race, living in a neighborhood that has more than 25% Black residents increases the odds of being obese (3), suggesting that elements of the neighborhood environment play a role in individual-level obesity. Neighborhood environment (physical, built, and social) has implications for health over the lifecourse (4) and the systematic disinvestment in some communities (i.e., redlining) contributes to disparities in health outcomes (5).

Williams and Collins suggest that racial residential segregation is the cornerstone on which Black–White disparities in health status have been built because it shapes socioeconomic opportunity structures, determines access to health promoting resources and services, and constrains individual choices that affect health risks (6). Racial segregation is a complex concept. Massey and Denton classified five key dimensions of segregation: evenness, exposure, concentration, centralization, and clustering (7). Here, we focus on two dimensions of segregation: evenness and exposure. Evenness is the measure of the differences in the distribution of the population, while exposure measures the potential for contact between the two groups. Concentration, centralization, and clustering all have a spatial component in their calculation which we do not assess here. Segregation creates differential exposure to critical resources that shape health trajectories (8) and empirical research has documented negative associations between segregation and health and mortality (9). Importantly, the effects of segregation are not borne only by Blacks of low-income status; due to discriminatory housing practices, “white flight” and experiences of interpersonal discrimination, Blacks are less able to reside in neighborhoods commensurate with their socioeconomic status, implying that they often live in economically and spatially non-distinct Black communities (10, 11). Blacks continue to be the most residentially segregated racial group with average neighborhood racial composition rates in 2010 similar to those in 1960 (10–12).

Examinations of the associations between segregation and weight status have led to mixed results (13–18), resulting in new research questions that consider the role of neighborhood characteristics as contributing risk factors to obesogenic environments (3, 19, 20). These studies suggest that neighborhood design plays an important role as a facilitator of, or barrier to, dietary behaviors. Limited access to supermarkets has been strongly associated with obesity risk and poorer dietary consumption, and the density of fast food restaurants in an area has been related to higher fat intake (21, 22). Correlations between characteristics (e.g., walkability, safety, food environment) of current residential environment and higher body mass index (BMI) have been established (23–25). Suggesting that neighborhoods perceived as safe, with an infrastructure that promotes physical activity (parks, sidewalks) and healthy diet (lower concentration of fast food restaurants, access to supermarkets with fresh fruits and vegetables) are less obesogenic (19, 26).

Racial segregation has been shown to influence obesity-related health behaviors of Black Americans. These studies indicate that Black neighborhoods have fewer supermarkets and a poorer selection of healthful dietary choices, such as fruits and vegetables, as compared to predominantly White neighborhoods (21, 27, 28). In contrast, these neighborhoods have a higher density of fast food restaurants per capita compared to White neighborhoods (28, 29). The positive association between racial residential segregation and BMI among Blacks persist even after adjusting for individual socioeconomic status (13). These studies provide evidence about distinct material disadvantages and resource deficits in predominately Black, compared to predominately White neighborhoods.

Large cities, such as Chicago, Philadelphia, and New York City have been the primary focus of studies on racial segregation and health promoting behaviors. There have been fewer studies examining the impact in medium-sized cities like Kansas City and St. Louis, MO, USA. The US state of Missouri is an ideal location to assess the role of segregation and food behaviors and environments in obesity. Missouri ranks 11th among states with the highest prevalence of obesity, with a population that is primarily non-Hispanic White (80%) and Black (11%) and high levels of segregation in its urban centers (St. Louis and Kansas City) (10–12) where most (78%) Black residents reside (30). Both St. Louis City (50%) and Kansas City (30%) have a substantial proportion of black residents. High levels of segregation in the two cities persist as a result of segregationist ideology perpetuated through urban planning initiatives, community building and reform movements and elite real estate (31, 32). In this context, we extend research that has established the relationship between racial residential segregation (evenness and exposure) and BMI (3, 13, 17, 18) to examine the mediating effect of food environment (individual and Census tract-level) and dietary behaviors at the individual level on the association between residential segregation and BMI among working non-Hispanic White and Black Missouri residents.

## Materials and Methods

### Data Sources and Study Population

**Figure 1:**
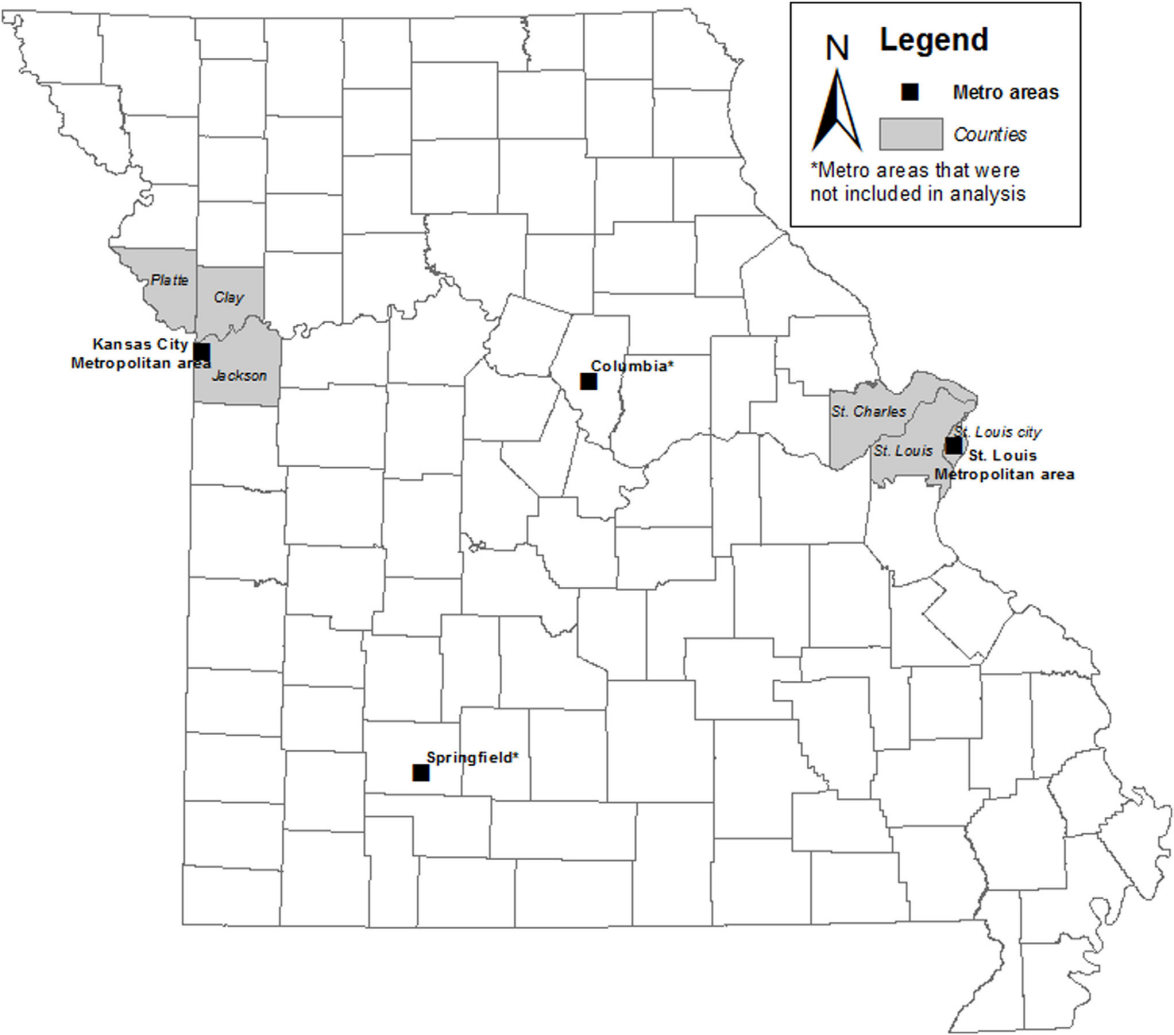
Map of Missouri by County with metropolitan areas sampled in the SHOW-ME study.

The Supports at Home and Work for Maintaining Energy Balance (SHOW-ME) study was a cross-sectional list-assisted random-digit-dialing telephone survey of 2,012 adults (aged 21–65) living and working (outside the home at one primary location for 20 or more hours per week at a site with at least five employees) in Missouri between April 2012 and April 2013 (33). Census tracts in six non-overlapping regions (Clay County, Jackson County, Platte County, St. Charles County, St. Louis County, and St. Louis City) from two large Missouri metropolitan areas (St. Louis, Kansas City) were used for the analysis. Figure 1 displays a map of Missouri by county indicating the locations of the metropolitan areas sampled in the SHOW-ME study.

Census tracts with a population density less than the 10th percentile of the population density of the study areas and those with more than 50% of inhabitants aged 15–24 years were excluded. A multistage, stratified sampling procedure was used to sample individuals across seven strata, including metro size (large vs. small), and within the large metro size, walkability (low, moderate, and high) (34), and racial/ethnic minority (low vs. high). The first eligible adult in each household that volunteered to participate was sampled. The response rate for interviews was 49%. Additional details on participant recruitment, exclusion and inclusion criteria, data collection, and sampling scheme have been previously published (33, 35).

For this analysis, we included only participants who identified solely as non-Hispanic White or non-Hispanic Black (*n* = 1,481) due to the small number of participants who identified as other or multiple races (*n* = 119) or with missing information on race (*n* = 16). Participants were also excluded if they had missing BMI data (*n* = 69). The analytic sample included 1,412 adults living in the Kansas City (*n* = 505) and St. Louis (*n* = 907) metro areas. We exclude data from Springfield and Columbia metropolitan areas from this analysis given the limited number of census tracts sampled (36 and 12, respectively) and low percentage of Black residents (4 and 10%, respectively). The majority (92%) of census tracts samples in the SHOW-ME study were in the St. Louis (356 census tracts) and Kansas City (237 census tracts) urban centers with a higher percentage of Black residents allowing for valid estimates of census tract-level segregation.

Census tract-level racial, economic, and educational data were obtained from the 2012 American Community Survey (ACS) (36). Food environment data were from the Centers for Disease Control and Prevention (CDC) (37). This study was approved by the Human Research Protections Office/Institutional Review Board of Washington University in St. Louis.

### Outcome Variable

Self-reported height and weight were collected on the SHOW-ME questionnaire. From these measures, each participant’s BMI was calculated and modeled as continuous for this analysis.

### Racial Segregation

We calculated two evenness (38) (dissimilarity index and entropy) and three exposure (38, 39) [isolation, correlation, and the local spatial segregation index (LSSI)] measures of Black–White residential segregation using 5-year estimates of non-Hispanic Black and non-Hispanic White populations from the 2012 ACS (36).

#### Evenness Segregation Measures

The dissimilarity index measures the percentage of a Census tract’s population that would have to move for each census tract to have the same racial composition as the overall county (38). The values range from 0 (complete integration) to 1 (complete segregation) and were calculated for each county (*n* = 6). Entropy is the weighted average deviation of each Census tract from the county’s racial/ethnic diversity, with values ranging from 0 (all census tracts have the same composition as the entire county) to 1 (all census tracts contain only one racial group). These values are summed across all Census tracts in the county to get the county’s entropy level (38).

#### Exposure Segregation Measures

Isolation measures the extent to which Blacks are exposed only to one another (38). Correlation is an adjustment of isolation to control for asymmetry as a result of the two racial groups not having the same proportion (38). The LSSI is calculated for each Census tract and measures the spatial segregation of Blacks in a Census tract from whites in the surrounding Census tracts (39). All three exposure measures range from 0 (complete integration) to 1 (complete segregation).

In sum, we have four county-level (dissimilarity index, entropy, isolation, correlation) measures of segregation and 1 Census tract-level (LSSI) of segregation; see Appendix in Supplementary Material for mathematical formulas of segregation measures.

### Neighborhood-Level Covariates

Participants’ home addresses were geocoded to determine their home Census tract. Neighborhood-level covariates were measured at the Census tract level. Percent non-Hispanic Black, percent with bachelor’s degree, and median household income were obtained from the 2012 ACS’s 5-year estimates. Walkability score was assigned by SHOW-ME investigators based on a *Z* score of population density (persons per square mile), retail density (retail establishments per square mile), and intersection intensity (number of 3-way or greater intersections) for each Census tract (34). High walkability was defined as above the 90th percentile, with moderate being in the 45th–90th percentiles, and low being less than the 45th percentile. Area level food environment was captured using the Centers for Chronic Disease Prevention modified retail food environment index (mRFEI) (37). The index provides a measure of the retail food environment of the Census tract, with a range of 0 (no food retailers that typically sell healthy food) to 100 (only food retailers that sell healthy food); a higher score indicates a higher percentage of healthful-food vendors (40). The definitions for healthy and less healthy food retailers are based on the Centers for Disease Control and Prevention definition (37). The mRFEI bases store classifications on the North American Industry Classification System codes. The mRFEI defines healthful-food retailers as supermarkets and larger grocery stores, supercenters, and produce stores (which include stands and markets that sell fruits and vegetables). The mRFEI defines less healthful-food retailers as convenience stores, smaller grocery stores with fewer than three employees, and fast food restaurants.

### Individual-Level Covariates

The SHOW-ME questionnaire assessed participant’s age, gender, race, education, as well as food environment and dietary habits. We evaluated individual-level food environment using a modified version of the access to healthy foods subscale (41). Comprised of three items (It is easy to purchase fresh fruits and vegetables in my neighborhood; the fresh produce in my neighborhood is of high quality; there is a large selection of fresh fruits and vegetables available in my neighborhood), items are answered on a four-point Likert scale and summed together to create the modified subscale value; higher values indicate lower access to healthy foods (Cronbach’s alpha = 0.91). The three items were also analyzed individually and were dichotomized for analysis (strongly agree/agree vs. disagree/strongly disagree). Individual dietary habits were assessed using self-reported daily servings of fruits, vegetables, and sugary items.

Income concordance was measured as the relationship between an individual’s household income and their home Census tract median household income. Median household income from the ACS was classified into income categories corresponding to those used to collect annual household income on the SHOW-ME survey. We compare if annual household income is in the same category as the median census tract income based on $10,000 increments. Participants could either be concordant with their Census tract (meaning their personal income was in the same income category as the Census tract’s median household income), discordant below (personal income was below the median household income), or discordant above (personal income was above the median household income).

Racial concordance was measured as the dichotomous relationship between individual-level race and the race of the majority of their home Census tract. If the individual’s race was the same as the race of 50% or greater of the census tract population, they were coded as concordant.

### Statistical Analyses

All statistical analyses were conducted using SAS/STAT version 9.4 (SAS Institute Inc., Cary, NC, USA). We conducted a multi-level mediation analysis to determine the extent to which the relationship between individual-level BMI and Census-level segregation is mediated by individual and Census tract-level food environments. To begin, the direct relationship between BMI and segregation was assessed using multi-level linear regression, accounting for clustering within Census tracts and counties (step 1). Multi-level models were used to account for the hierarchical nature of the data (individuals, Census tracts, counties). Next, the association between each of the segregation measures significant in step 1 and the potential mediators (individual-level dietary habits and food environment, and Census-level food environment associated with BMI) were tested with multi-level linear regression (step 2). If a mediator had a significant association with a particular segregation measure then they were both entered into a full, multi-level model to test their joint association with BMI (step 3). If the segregation measure became insignificant and the mediator remained significant, the relationship between racial segregation and BMI was determined to be fully mediated. If both the segregation measure and the predictor remained significant, the relationship was determined to be partially mediated. If the mediator did not affect the relationship between racial segregation and BMI (it remained significant) and the mediator itself becomes insignificant, no mediation occurs. We examined mediation in adjusted multi-level regression models controlling for important confounding factors (walkability of Census tract, individual race concordance with Census tract majority, and individual income concordance with Census tract median household income).

## Results

Table 1 displays the frequency and overall percent for all categorical variables, as well as the mean and SD for all continuous variables. Based on individual-level data, the sample is primarily female (68%), White (62%), has an annual household income of $40,000 or more (68%), and has a college degree or higher education (52%). Most participants reported easy access to (84%), and a large selection of (80%) fresh produce that is high quality (81%).

**Table 1:** Demographics and area level descriptors of the sample (*n* = 1,412).

	**Overall**		**Clay County (*n* = 68)**		**Jackson County (*n* = 410)**		**Platte County (*n* = 27)**		**St. Charles County (*n* = 140)**		**St. Louis County (*n* = 270)**		**St. Louis City (*n* = 497)**	
							
**Individual level—categorical**	***N***	**%**	***N***	**%**	***N***	**%**	***N***	**%**	***N***	**%**	***N***	**%**	***N***	**%**

Female	963	68.2	43	63.2	281	68.5	22	81.5	89	63.6	187	69.3	341	68.6
African-American	534	37.8	4	5.9	174	42.4	3	11.1	6	4.3	82	30.4	265	53.3
Income (*n* = 1,338)														
<$40K	421	31.5	12	19.4	154	39.1	2	8.0	12	9.0	40	15.9	201	42.5
$40K–$70K	399	29.8	17	27.4	137	34.8	9	36.0	34	25.6	68	27.1	134	28.3
$70K+	518	38.7	33	53.2	103	26.1	14	56.0	87	65.4	143	57.0	138	29.2
Education (*n* = 1,411)														
Less than college degree	674	47.8	30	44.1	205	50.1	10	37.0	62	44.3	100	37.0	267	53.7
College degree	455	32.3	29	42.7	131	32.0	12	44.4	46	32.9	104	38.5	133	26.8
Graduate degree	282	20.0	9	13.2	73	17.9	5	18.5	32	22.9	66	24.4	97	19.5
Easy to buy fresh produce (*n* = 1,411)	1,180	83.6	58	85.3	325	79.3	26	96.3	113	80.7	253	93.7	405	81.7
High quality of produce (*n* = 1,396)	1,136	81.4	57	83.8	315	77.6	25	92.6	111	79.9	241	89.9	387	79.3
Large selection of produce (*n* = 1,403)	1,121	79.9	59	86.8	306	75.0	25	92.6	106	75.7	241	89.3	384	78.4
Race concordant	1,172	83.0	64	94.1	312	76	24	88.9	134	95.7	236	87.4	402	80.9
Income concordance														
<Census tract median	196	14.7	17	27.4	60	15.2	11	44.0	27	20.3	44	17.5	37	7.8
=Census tract median	602	45.0	29	46.8	173	43.9	14	56.0	78	58.7	93	37.1	215	45.5
>Census tract median	540	40.4	16	25.8	161	40.9	0	0.0	28	21.1	114	45.4	221	46.7
Walkability of home Census tract														
Low walkability	543	38.5	68	100.0	168	41.0	27	100.0	99	70.7	160	59.3	21	4.2
Moderate walkability	348	24.7	0	0.0	144	35.1	0	0.0	22	15.7	99	36.7	83	16.7
High walkability	521	36.9	0	0.0	98	23.9	0	0.0	19	13.6	11	4.1	393	79.1

**Individual level—continuous**	**Mean**	**SD**	**Mean**	**SD**	**Mean**	**SD**	**Mean**	**SD**	**Mean**	**SD**	**Mean**	**SD**	**Mean**	**SD**

Echeverria scale	5.67	2.14	5.71	1.87	6.05	2.26	4.37	1.64	5.74	2.42	5.09	1.78	5.72	2.11
Daily intake of vegetables (*n* = 1,408)	1.87	1.56	2.05	1.16	1.82	1.55	2.03	1.24	1.85	1.23	1.97	1.66	1.84	1.65
Daily intake of fruits (*n* = 1,409)	1.55	1.37	1.48	1.61	1.48	1.42	1.29	1.23	1.50	1.28	1.68	1.36	1.57	1.34
Daily intake of sugars (*n* = 1,411)	1.63	2.15	1.78	1.91	1.71	2.69	1.16	1.09	1.51	2.47	1.31	1.26	1.79	1.98
Age (*n* = 1,405)	48.52	10.89	45.93	9.55	48.15	11.13	46.44	9.98	46.45	10.82	50.31	10.25	47.05	11.07
Body mass index	28.60	6.66	27.39	5.59	28.99	6.89	26.64	4.87	27.15	5.11	28.03	5.77	29.3	7.37

	**Overall (*n* = 103)**		**Clay County (*n* = 4)**		**Jackson County (*n* = 42)**		**Platte County (*n* = 2)**		**St. Charles County (*n* = 9)**		**St. Louis County (*n* = 13)**		**St. Louis City (*n* = 33)**	
**Census tract level – continuous**	**Mean**	**SD**	**Mean**	**SD**	**Mean**	**SD**	**Mean**	**SD**	**Mean**	**SD**	**Mean**	**SD**	**Mean**	**SD**

Percent African-American/Black	42.69	35.43	2.96	2.01	42.68	31.97	4.46	0.94	4.84	3.59	35.26	31.87	63.09	34.12
Percent bachelor’s degree	17.68	10.58	19.71	6.41	16.09	11.44	23.22	5.36	24.69	6.72	23.16	8.96	15.04	10.18
Modified retail food environment index (*n* = 71)	8.41	7.32	8.00	–	8.30	8.74	5.00	7.07	2.50	3.54	14.40	6.13	7.10	5.53
Local spatial segregation index	0.95	0.07	0.84	0.04	0.98	0.02	0.64	0.20	0.91	0.03	0.97	0.02	0.94	0.05
Median household income ($)	43,032	20,791	65,914	16,744	37,808	16,952	71,417	1,964	69,542	18,618	58,269	20,019	31,956	13,275

	**Overall (*n* = 6)**		**Clay County**		**Jackson County**		**Platte County**		**St. Charles County**		**St. Louis County**		**St. Louis City**	
		
**County level – continuous**	**Mean**	**SD**	**Value**		**Value**		**Value**		**Value**		**Value**		**Value**	

Percent African-American/Black	19.92	18.69	5.37		26.11		6.32		4.66		24.68		52.41	
Dissimilarity index	0.49	0.18	0.34		0.62		0.38		0.27		0.70		0.65	
Entropy	0.24	0.20	0.09		0.38		0.07		0.01		0.46		0.44	
Isolation index	0.38	0.32	0.09		0.59		0.11		0.07		0.64		0.77			
Correlation ratio	0.27	0.25	0.04		0.45		0.05		0.02		0.53		0.52	

On average, Census tracts in Jackson County and St. Louis City had the highest percentage of African-American or Black residents (an average of 43 and 63%, respectively). Census tracts in St. Louis City had the lowest average median household ($31,956) and those in Platte County had the highest ($71,417). The average percentage of healthy food retailers in a Census tract, as measured by the mRFEI, was 8% (SD = 7%). Over a quarter (28%) of the Census tracts in the sample are “food deserts” (mRFEI = 0; data not shown).

The majority of participants lived in Jackson County (29%) and St. Louis City (35.2%). The extent of segregation of the counties in which participants lived covered a broad range. The dissimilarity index ranged from mostly integrated (0.27, St. Charles County) to extremely segregated (0.70, St. Louis County) as did the isolation index, ranging from 0.07 (St. Louis City) to 0.77 (St. Charles County). There is similar variability across the other measures of segregation (Table 1).

### Unadjusted Analysis

There was a significant (*p* < 0.05) relationship between BMI and all of the segregation measures examined (Table 2). An increase in Census-level racial segregation was associated with a significant increase in individual BMI (Table 2). The relationship between BMI and county-level isolation and correlation was fully mediated by mRFEI. As the percentage of healthful-food vendors increased, BMI decreased. The effect of mRFEI was consistent across these two models with a one percentage point increase yielding a decrease in BMI of 0.09 kg/m^2^ (SE 0.04; *p* = 0.049).

**Table 2:** Multi-level regression estimates of association of residential racial segregation and body mass index (BMI), with and without mediation by food environment.

Unmediated models—segregation and BMI (*n* = 1,412)							

	β	SE	*p*-Value				
Evenness							
Dissimilarity index	4.28	2.17	0.049				
Entropy	4.20	1.87	0.025				
Exposure							
Isolation index	2.93	1.07	0.006				
Correlation ratio	3.57	1.49	0.017				
Local spatial segregation index (LSSI)	7.48	3.63	0.040				

**Mediated models—segregation and BMI with mediators: food environment (mRFEI), diet**							

	**Racial segregation**			**Mediator**			
	**β**	**SE**	***p*-Value**	**Mediator**	**β**	**SE**	***p*-Value**

Evenness							
Dissimilarity index (*n* = 976)	2.41	3.53	0.494	mRFEI	−0.08	0.05	0.073
Entropy (*n* = 976)	3.11	2.99	0.299	mRFEI	−0.09	0.05	0.052
Exposure							
Isolation index (*n* = 976)	2.60	1.67	0.119	mRFEI	−0.09	0.04	0.050
Correlation ratio (*n* = 976)	2.74	2.40	0.254	mRFEI	−0.09	0.04	0.049
LSSI (*n* = 1,403)	6.43	3.68	0.081	Large	−1.39	0.45	0.002
(*n* = 1,412)	6.13	3.65	0.094	Echeverria	0.27	0.08	0.002
(*n* = 1,408)	6.61	3.61	0.068	Vegetables	−0.39	0.11	<0.001

*mRFEI is the modified retail food environment index; a higher score indicates a higher percentage of healthful-food vendors. Echeverria is the modified access to health foods subscale. Large is large selection of produce. Vegetables is daily intake of vegetables*.

The Census tract-level LSSI’s effect on BMI was fully mediated by several individual-level food environment and dietary choices (Table 2). The LSSI pathway to BMI becomes insignificant when the modified access to healthy foods subscale, large selection of produce, or daily serving of vegetables are added to the multi-level regression model. Increased disadvantage in accessing healthy foods was associated with a significant increase in BMI (β = 0.27, SE 0.08, *p* = 0.002), and those who reported a large selection of produce had a significant decrease in BMI (β = −1.39, SE 0.45, *p* = 0.002). An increase of one serving of vegetables per day was associated with a significant decrease in BMI (β = −0.39, SE 0.11, *p* < 0.001).

### Adjusted Analyses

Models were adjusted for several covariates: the Census tract’s walkability, and racial and income concordance between an individual and their home Census tract (Table 3). After adjusting for these covariates, significant associations (*p* < 0.05) were found between BMI and several segregation measures (entropy, isolation index, correlation ratio, LSSI). Similar to the unadjusted models, adjusted analyses show a significant association between increases in Census-level racial segregation and individual BMI. An increase in racial segregation was associated with an increase in BMI ranging from 4.11 (correlation ratio) to 7.76 kg/m^2^ (LSSI).

**Table 3:** Race, income, and walkability adjusted regression estimates of association of residential racial segregation and body mass index (BMI), with and without mediation by food environment.

Adjusted models—segregation and BMI, covariates																					

	Dissimilarity index			Entropy			Isolation index			Correlation ratio			Local spatial segregation index (LSSI)								
	β	SE	*p*	β	SE	*p*	β	SE	*p*	β	SE	*p*	β	SE	*p*						
Segregation	4.91	2.6	0.061	4.86	2.3	0.032	3.53	1.3	0.005	4.11	1.8	0.025	7.76	3.8	0.041						
Walkability^a^																					
Low	0.26	0.7	0.700	0.27	0.7	0.696	0.26	0.7	0.688	0.27	0.7	0.690	−0.63	0.6	0.284						
Moderate	0.79	0.7	0.239	0.78	0.7	0.242	0.76	0.6	0.238	0.76	0.7	0.250	0.21	0.7	0.756						
Race concordance^b^	−0.88	0.5	0.078	−0.88	0.5	0.079	−0.88	0.5	0.078	−0.88	0.5	0.081	−0.94	0.5	0.059						
Income^c^																					
Below	0.76	0.6	0.167	0.76	0.6	0.166	0.77	0.6	0.165	0.77	0.6	0.165	0.75	0.6	0.172						
Above	−1.00	0.4	0.013	−1.01	0.4	0.013	−1.02	0.4	0.011	−1.01	0.4	0.012	−0.99	0.4	0.014						

**Adjusted models—segregation and BMI, mediator: food environment and diet, covariates**																					

	**Dissimilarity index and mRFEI**			**Entropy and mRFEI**			**Isolation index and mRFEI**			**Correlation ratio and mRFEI**			**LSSI and large selection of produce**			**LSSI and Echeverria**			**LSSI and daily vegetable intake**		
	**β**	**SE**	***p***	**β**	**SE**	***p***	**β**	**SE**	***p***	**β**	**SE**	***p***	**β**	**SE**	***p***	**β**	**SE**	***p***	**β**	**SE**	***p***

Segregation	2.12	3.8	0.579	2.86	3.3	0.392	2.82	1.97	0.152	2.48	2.7	0.360	6.88	3.9	0.075	6.59	3.8	0.086	6.91	3.8	0.067
Mediator	−0.08	<0.1	0.072	−0.09	0.05	0.056	−0.09	0.04	0.036	−0.09	0.05	0.054	−1.27	0.5	0.005	0.23	0.1	0.007	−0.35	0.1	0.003
Walkability^a^																					
Low	−0.28	0.8	0.732	−0.27	0.8	0.740	−0.21	0.8	0.804	−0.26	0.8	0.751	−0.72	0.6	0.231	−0.67	0.6	0.258	−0.64	0.6	0.271
Moderate	0.65	0.8	0.389	0.63	0.7	0.400	0.62	0.7	0.395	0.62	0.7	0.404	0.07	0.7	0.913	0.16	0.7	0.806	0.17	0.7	0.791
Race concordance^b^	−1.08	0.6	0.067	−1.07	0.6	0.071	−1.03	0.6	0.080	−1.06	0.6	0.073	−0.86	0.5	0.086	−0.84	0.5	0.094	−0.88	0.5	0.077
Income^c^																					
Below	1.35	0.7	0.053	1.37	0.7	0.050	1.40	0.7	0.045	1.37	0.7	0.049	0.74	0.6	0.178	0.69	0.6	0.208	0.74	0.6	0.178
Above	−0.65	0.5	0.172	−0.66	0.5	0.166	−0.67	0.5	0.157	−0.66	0.5	0.165	−1.02	0.4	0.012	−1.01	0.4	0.013	−0.94	0.4	0.021

*^a^Reference is high walkability of home Census tract*.

*^b^Reference is race discordant with Census tract*.

*^c^Income Concordance: reference is concordant with Census tract median household income*.

*mRFEI is the modified retail food environment index; a higher score indicates a higher percentage of healthful-food vendors. Echeverria is the modified access to health foods subscale*.

When mRFEI was added to the adjusted models for entropy, isolation index, and correlation ratio, the segregation measures became insignificant. For the adjusted models for entropy and correlation, mRFEI did not remain significant (no mediation). For the adjusted models for isolation, mRFEI fully mediated their relationship with BMI. The mediator’s effect remained consistent across models, with a 0.9-kg/m^2^ decrease in BMI for every percentage increase in healthy food retailers. In adjusted models, the LSSI’s effect on BMI was fully mediated by the modified access to healthy foods subscale, large selection of produce, and daily serving of vegetables. In all three models, an increase in access, availability or consumption of healthy foods was associated with a significant decrease in BMI.

## Discussion

**Figure 2:**
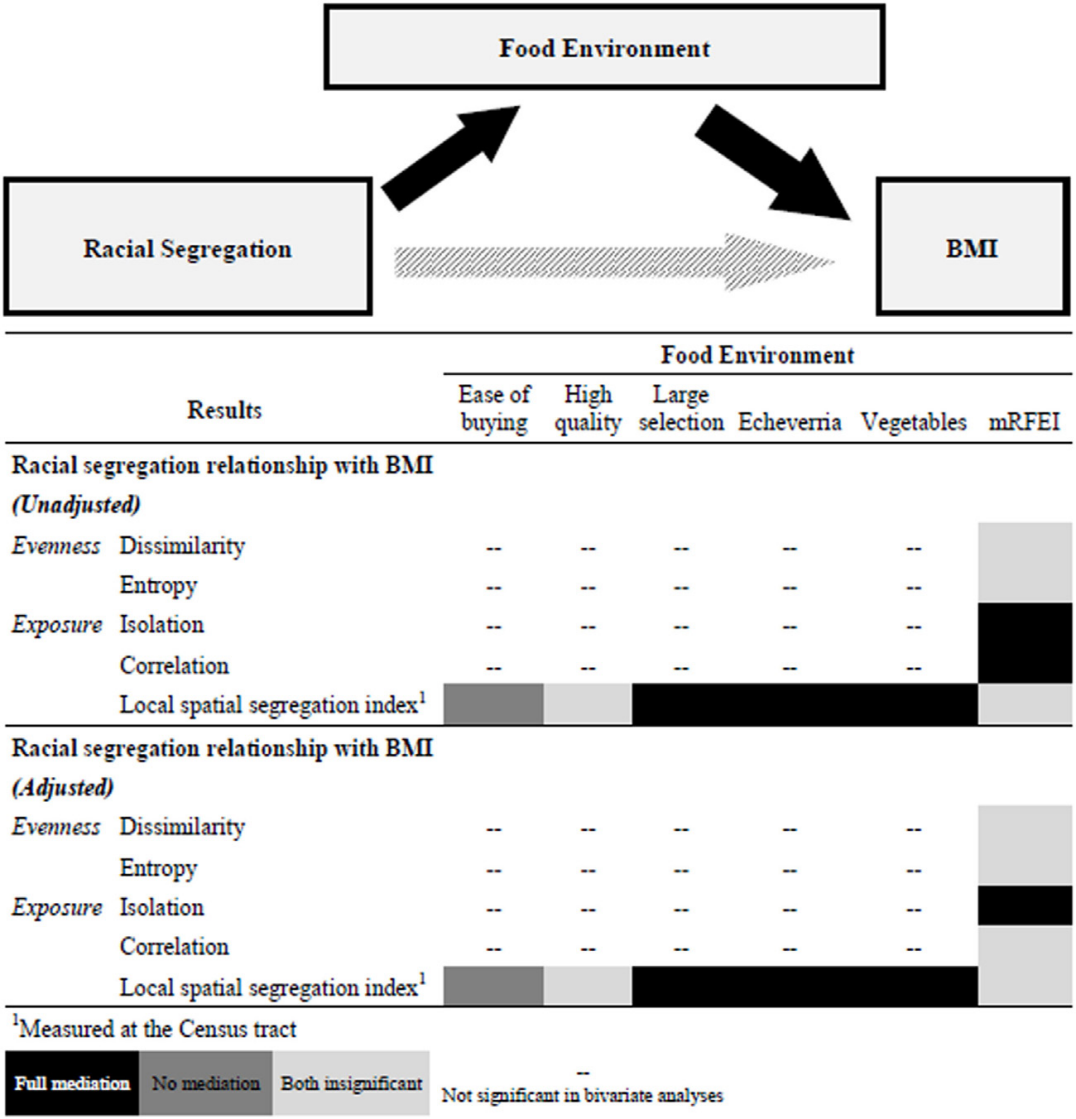
Mediation pathway and Gantt chart summary of results. Note: mRFEI is the modified retail food environment index; a higher score indicates a higher percentage of healthful-food vendors. Easy of buying is easy to by fresh produce. High quality is high quality of produce. Large selection is large selection of produce. Echeverria is the modified access to health foods subscale. Vegetables is daily intake of vegetables.

There is some evidence to suggest a relationship between racial residential segregation (evenness and exposure) and BMI (3, 13, 17, 18). What is not clear about this relationship is whether the food environment (individual and Census tract-level) and dietary behaviors at the individual level mediate the relationship between segregation and BMI. In the present study, we examined if food environment and dietary behaviors mediate the relationship between racial residential segregation and BMI. In unadjusted analyses, we find that for evenness measures of segregation, adding food environment to the models makes both variables insignificant. Among county-level exposure measures of segregation, area food environment fully mediates this relationship and individual food environment fully mediates the Census tract-level exposure measure of segregation. These findings from the unadjusted analyses hold in the adjusted models except for correlation which behaves like the evenness measures of segregation (both variables are insignificant) in adjusted analyses (Figure 2). The dissimilarity index was no longer significant in adjusted models. This potentially means that, even in the most segregated areas, the impact of segregation on obesity could be mitigated by changing the retail food environment in the areas experiencing higher obesity prevalence. However, the placement of grocery stores in food deserts may not necessarily lead to improvements in dietary behaviors or BMI (42, 43). This increase in access will only be effective if coupled with policies and programs that make healthy choices affordable and train residents how to prepare healthy meals.

The examination of food environment at both the individual and Census tract-level is a major strength of the study. In the adjusted models we find that food environment mediates at one level below the level where segregation is measured and there are no associations between segregation measured at the county level and food environment on the individual level (two levels apart). The examination of two domains of segregation: evenness and exposure shows differences in the mediation across domains. Our findings suggest that when evenness measures of segregation (dissimilarity index and entropy) and food environment (mRFEI) are simultaneously in models predicting BMI neither is a significant predictor. However, when segregation is measured as exposure, food environment mediates the relationship between segregation and BMI.

Given the multi-level nature of our data (individuals, Census tracts, counties) the literature is mixed in terms of how to handle mediation in this context. We used a standardized approach (44–46) for testing mediation in heterarchical linear regression models. The multi-level nature of our regression models and the examination if mediation still exists while adjusting for important confounders is an important extension to standard mediation analysis; we compared the results of these adjusted analyses to our unadjusted models. We used a concordance approach when adjusting for variables that are significant at the individual and Census tract-level (i.e., race and income) to account for those who are discordant with the majority race in an area who may experience an advantage or disadvantage being in a neighborhood where you were not a member of the majority race or income level.

It is important to interpret these results in light of several study limitations. The primary outcome BMI is based on self-reported data. Although people sometimes underestimate their weight, their estimates are generally accurate (47); however, the lack of objective measurement of height and weight is a major study limitation. Underestimation of weight will attenuate BMI calculations potentially biasing the estimates. However, given the variability of BMI in our sample, we believe our models are robust despite the self-reported nature of the height and weight data. While the multi-level nature of our data is a strength of this work, it is important to note difference in data at each level. All individual-level data are self-reported and all area level (county, Census tract) is based on objective Census and retail food environment data. As with all secondary data analysis, the primary survey design and data collection was not collected for the purpose of this analysis. We control for the study design sampling of Census tracts based on walkability and racial composition by controlling for these factors in adjusted models. Our results are not generalizable to non-working populations. The cross-sectional nature of the study design does not allow for causal inferences, rather we examine associations between variables. Our results highlight this as in some models both the primary predictor (segregation) and the mediator (food environment) are insignificant when modeled simultaneously. As with all cross-sectional studies we are unable to determine whether the outcome (BMI) followed the exposure (segregation) and mediator (food environment and food behaviors) in time as all three variables were measured at the same time point.

Despite these limitations the results provide strong implications that food environment mediates the association between residential segregation and BMI. However, there are several important things to consider when examining these relationships using statistical models. The domain of segregation being measured and the level at which each of the variables are measured—predictor (segregation), mediator (food environment), and outcome (BMI)—have implications for mediating effects. This analysis provides additional evidence of the relationship between residential segregation and food environment with changes in the racial composition of neighborhoods being a strong predictor of changes in the food environment. Our results also support the assertion that it is the lack of resources for healthy food in racially segregated areas that affects BMI, even when adjusting for individual dietary behaviors. Future work should continue to examine modifiable mediators in the relationship between segregation and BMI and develop necessary interventions, programs, and policies that modify these mediators to examine if this can reduce the disproportionate burden of obesity among non-Hispanic Blacks.

## Author Contributions

MG helped conceive the study, develop the analysis plan, conduct data analysis, and draft the manuscript. SL conducted the data analysis and helped draft the Methods and Results sections of the manuscript. CA helped conceive the study, data analysis plan, and draft the manuscript. LD helped conceive the study and draft the manuscript. JH was Principal Investigator of the SHOW-ME study, he helped conceive the current study and draft the manuscript. All authors have approved the final version of the manuscript.

## Conflict of Interest Statement

The authors declare that the research was conducted in the absence of any commercial or financial relationships that could be construed as a potential conflict of interest.

## References

[1] Drewnowski A Specter SE. Poverty and obesity: the role of energy density and energy costs. Am J Clin Nutr (2004) 79(1):6–16. 10.1093/ajcn/79.1.6 14684391

[2] Ogden CL Carroll MD Kit BK Flegal KM. Prevalence of childhood and adult obesity in the United States, 2011-2012. JAMA (2014) 311(8):806–14. 10.1001/jama.2014.732 24570244 PMC4770258

[3] Boardman JD Saint Onge JM Rogers RG Denney JT. Race differentials in obesity: the impact of place. J Health Soc Behav (2005) 46(3):229–43. 10.1177/002214650504600302 16259146 PMC3171451

[4] Diez Roux AV Mair C. Neighborhoods and health. Ann N Y Acad Sci (2010) 1186:125–45. 10.1111/j.1749-6632.2009.05333.x 20201871

[5] Acevedo-Garcia D Lochner KA Osypuk TL Subramanian SV. Future directions in residential segregation and health research: a multilevel approach. Am J Public Health (2003) 93(2):215. 10.2105/AJPH.93.2.215 12554572 PMC1447719

[6] Williams DR Collins C. Racial residential segregation: a fundamental cause of racial disparities in health. Public Health Rep (2001) 116(5):404. 10.1016/S0033-3549(04)50068-7 12042604 PMC1497358

[7] Massey DS Denton NA The dimensions of residential segregation. Soc Forces (1988) 67(2):281 10.1093/sf/67.2.281

[8] Laveist TA Wallace JM Health risk and inequitable distribution of liquor stores in African American neighborhoods. Soc Sci Med (2000) 51(4):613–7. 10.1016/S0277-9536(00)00004-6 10868674

[9] Subramanian SV Acevedo-Garcia D Osypuk TL. Racial residential segregation and geographic heterogeneity in black/white disparity in poor self-rated health in the US: a multilevel statistical analysis. Soc Sci Med (2005) 60(8):1667–79. 10.1016/j.socscimed.2004.08.040 15686800

[10] Logan JR The Persistence of Segregation in the 21st Century Metropolis. City Community (2013) 12(2):160–8. 10.1111/cico.12021 PMC3859616 24348102

[11] Massey DS Denton NA American apartheid: segregation and the making of the underclass. AJS (1990) 96(2):329–57. 10.1086/229532

[12] Gordon CC Mapping Decline: St. Louis and the Fate of the American City. Philadelphia, PA: University of Pennsylvania Press (2008).

[13] Chang VW. Racial residential segregation and weight status among US adults. Soc Sci Med (2006) 63(5):1289–303. 10.1016/j.socscimed.2006.03.049 16707199

[14] Cozier YC Yu J Coogan PF Bethea TN Rosenberg L Palmer JR. Racism, segregation, and risk of obesity in the black women’s health study. Am J Epidemiol (2014) 179(7):875–83. 10.1093/aje/kwu004 24585257 PMC3969538

[15] Kershaw KN Albrecht SS. Metropolitan-level ethnic residential segregation, racial identity, and body mass index among U.S. Hispanic adults: a multilevel cross-sectional study. BMC Public Health (2014) 14(1):283. 10.1186/1471-2458-14-283 24669799 PMC4004511

[16] Kershaw KN Albrecht SS Carnethon MR. Racial and ethnic residential segregation, the neighborhood socioeconomic environment, and obesity among blacks and Mexican Americans. Am J Epidemiol (2013) 177(4):299–309. 10.1093/aje/kws372 23337312 PMC3566709

[17] Corral I Landrine H Zhao L. Residential segregation and obesity among a national sample of Hispanic adults. J Health Psychol (2014) 19(4):503–8. 10.1177/1359105312474912 23460679

[18] Corral I Landrine H Hao Y Zhao L Mellerson JL Cooper DL. Residential segregation, health behavior and overweight/obesity among a national sample of African American adults. J Health Psychol (2012) 17(3):371–8. 10.1177/1359105311417191 21844135

[19] Boehmer TK Hoehner CM Deshpande AD Brennan Ramirez LK Brownson RC. Perceived and observed neighborhood indicators of obesity among urban adults. Int J Obes (Lond) (2007) 31(6):968–77. 10.1038/sj.ijo.0803531 17224932

[20] Wang Y Beydoun MA The obesity epidemic in the United States – gender, age, socioeconomic, racial/ethnic, and geographic characteristics: a systematic review and meta-regression analysis. Epidemiol Rev (2007) 29(1):6–28. 10.1093/epirev/mxm007 17510091

[21] Morland K Diez Roux AV Wing S. Supermarkets, other food stores, and obesity: the atherosclerosis risk in communities study. Am J Prev Med (2006) 30:333–9. 10.1016/j.amepre.2005.11.003 16530621

[22] Maddock J The relationship between obesity and the prevalence of fast food restaurants: state-level analysis. Am J Health Promot (2004) 19(2):137–43. 10.4278/0890-1171-19.2.137 15559714

[23] Rundle A Neckerman KM Freeman L Lovasi GS Purciel M Quinn J Neighborhood food environment and walkability predict obesity in New York City. Environ Health Perspect (2009) 117(3):442–7. 10.1289/ehp.11590 19337520 PMC2661915

[24] Inagami S Cohen DA Brown AF Asch SM. Body mass index, neighborhood fast food and restaurant concentration, and car ownership. J Urban Health (2009) 86(5):683–95. 10.1007/s11524-009-9379-y 19533365 PMC2729867

[25] Gibson DM. The neighborhood food environment and adult weight status: estimates from longitudinal data. Am J Public Health (2011) 101(1):71–8. 10.2105/AJPH.2009.187567 21088263 PMC3000723

[26] Sallis JF Glanz K. Physical activity and food environments: solutions to the obesity epidemic. Milbank Q (2009) 87(1):123–54. 10.1111/j.1468-0009.2009.00550.x 19298418 PMC2879180

[27] Powell LM Slater S Mirtcheva D Bao Y Chaloupka F Food store availability and neighborhood characteristics in the United States. Prev Med (Baltim) (2007) 44(3):189–95. 10.1016/j.ypmed.2006.08.008 16997358

[28] Baker EA Schootman M Barnidge E Kelly C. The role of race and poverty in access to foods that enable individuals to adhere to dietary guidelines. Prev Chronic Dis (2006) 3(3):1–11. 16776877 PMC1636719

[29] Block JP Scribner RA Desalvo KB. Fast food, race/ethnicity, and income: a geographic analysis. Am J Prev Med (2004) 27(3):211–7. 10.1016/S0749-3797(04)00139-4 15450633

[30] Health Equity Series: African American Health Disparities in Missouri. St. Louis, MO: Missouri Foundation for Health (2013). Available from: https://mffh.org/wordpress/wp-content/uploads/2016/04/13AfrAmDisparities.pdf

[31] Gotham KF Urban space, restrictive covenants and the origins of racial residential segregation in a US city, 1900–50. Int J Urban Reg Res (2000) 24:616–33. 10.1111/1468-2427.00268

[32] Tighe JR Ganning JP The divergent city: unequal and uneven development in St. Louis. Urban Geogr (2015) 36(5):654–73. 10.1080/02723638.2015.1014673

[33] Hoehner CM Budd EL Marx CM Dodson EA Brownson RC. Development and reliability testing of the worksite and energy balance survey. J Public Health Manag Pract (2013) 19(3 Suppl 1):S105–13. 10.1097/PHH.0b013e3182849f21 23529049 PMC4039347

[34] Frank LD Sallis JF Saelens BE Leary L Cain K Conway TL The development of a walkability index: application to the neighborhood quality of life study. Br J Sports Med (2010) 44:924–33. 10.1136/bjsm.2009.058701 19406732

[35] Yang L Hipp JA Adlakha D Marx CM Tabak RG Brownson RC. Choice of commuting mode among employees: do home neighborhood environment, worksite neighborhood environment, and worksite policy and supports matter? J Transp Health (2015) 2(2):212–8. 10.1016/j.jth.2015.02.003 26085979 PMC4465081

[36] United States Census Bureau. 2008 – 2012 American Community Survey 5-Year Estimates.

[37] Centers for Disease Control and Prevention. Modified Retail Food Environment Index. Obes State Indic Reports (2011).

[38] Iceland J Weinberg DH Steinmetz E Racial and Ethnic Residential Segregation in the United States: 1980-2000. Washington, DC: U.S. Government Printing Office (2002).

[39] Wong DWS Modeling local segregation: a spatial interaction approach. Geogr Environ Model (2002) 6(1):81–97. 10.1080/13615930220127305

[40] Gustafson AA Lewis S Wilson C Jilcott-Pitts S. Validation of food store environment secondary data source and the role of neighborhood deprivation in Appalachia, Kentucky. BMC Public Health (2012) 12(1):688. 10.1186/1471-2458-12-688 22914100 PMC3491041

[41] Echeverria SE Diez-Roux AV Link BG. Reliability of self-reported neighborhood characteristics. J Urban Health (2004) 81(4):682–701. 10.1093/jurban/jth151 15466849 PMC3455923

[42] Elbel B Moran A Dixon LB Kiszko K Cantor J Abrams C Assessment of a government-subsidized supermarket in a high-need area on household food availability and children’s dietary intakes. Public Health Nutr (2015) 18(15):1–10. 10.1017/S1368980015000282 PMC10271373 25714993

[43] Cummins S Flint E Matthews SA. New neighborhood grocery store increased awareness of food access but did not alter dietary habits or obesity. Health Aff (Millwood) (2014) 33(2):283–91. 10.1377/hlthaff.2013.0512 24493772 PMC4201352

[44] Zhang Z Zyphur MJ Preacher KJ Testing multilevel mediation using hierarchical linear models: problems and solutions. Acad Manag Annu Meet Proc (2008) 8(1):1–6. 10.1177/1094428108327450

[45] Preacher KJ Zhang Z Zyphur MJ Multilevel structural equation models for assessing moderation within and across levels of analysis. Psychol Methods (2015) 20(4):1–17. 10.1037/met0000052 26651982

[46] Lachowicz MJ Sterba SK Preacher KJ Investigating multilevel mediation with fully or partially nested data. Gr Process Intergr Relations (2015) 18(3):274–89. 10.1177/1368430214550343

[47] McAdams MA Van Dam RM Hu FB. Comparison of self-reported and measured BMI as correlates of disease markers in US adults. Obesity (2007) 15(1):188–96. 10.1038/oby.2007.504 17228047

