# Supplementary material for: How Segregation Makes Us Fat: Food Behaviors and Food Environment as Mediators of the Relationship Between Residential Segregation and Individual Body Mass Index
Source: Front Public Health. 2018 Mar 29;6:92. doi: 10.3389/fpubh.2018.00092 (PMC5884945; doi:10.3389/fpubh.2018.00092)
Supplement: Supplementary file 1 [file data_sheet_1.docx]

Appendix: Calculation of Segregation Measures

**Definitions**

| w_i_ | Number of Whites in a Census tract |  | t_i_ | Total population of Whites and Blacks in a Census tract |  |
| --- | --- | --- | --- | --- | --- |
| b_i_ | Number of Blacks in a Census tract |  | T | Total population of Whites and Blacks in a county |  |
|  | Total Whites in county |  | p_i_ | Proportion of Blacks to total population in a Census tract |  |
| B | Total Blacks in county |  | P | Proportion of Blacks to total population in a county |  |

**Evenness**

Dissimilarity Index – the percentage of a group’s population that would have to change residence for each Census tract to have the same percentage

$$D= \frac{1}{2}\sum_{i} \left| \frac{w_{i}}{W}-\frac{b_{i}}{B} \right|$$

Entropy Index – the weighted average deviation of each areal unit from the county’s entropy or racial and ethnic diversity, which is greatest when each group is equally represented in the county

$$Entropy=\sum_{i} \frac{t_{i}(E-E_{i})}{ET}$$

Where $E=Pln\left( \frac{1}{P} \right)+\left( 1-P \right)ln\left( \frac{1}{1-P} \right)$

and $E_{i}=p_{i}ln\left( \frac{1}{p_{i}} \right)+\left( 1-p_{i} \right)ln\left( \frac{1}{1-p_{i}} \right)$

**Exposure**

Local Spatial Segregation Index – lack of potential for interaction between blacks in a certain Census tract and whites

$$S_{i*bw}=1-\frac{b_{i}\sum_{j} c_{ij}w_{j}}{b_{i}\sum_{j} w_{j}}$$

Isolation – the extent to which minority members are exposed only to one another

$$Isolation=I=\sum_{i} \left( \frac{b_{i}}{B} \right)\left( \frac{b_{i}}{t_{i}} \right)$$

Correlation ratio – adjustment of the isolation index to control for asymmetry of the isolation index (I)

$$\eta^{2}=\frac{\left( I-P \right)}{\left( 1-P \right)}$$
